# Dishevelled1-3 contribute to multidrug resistance in colorectal cancer via activating Wnt/β-catenin signaling

**DOI:** 10.18632/oncotarget.23253

**Published:** 2017-12-14

**Authors:** Kun Zhang, Minhui Li, Houyi Huang, Linpeng Li, Jie Yang, Li Feng, Junjie Gou, Mengju Jiang, Liaotian Peng, Linyi Chen, Ting Li, Ping Yang, Yuhan Yang, Yuanyuan Wang, Quekun Peng, Xiaozhen Dai, Tao Zhang

**Affiliations:** ^1^ School of Biomedical Sciences, Chengdu Medical College, Chengdu, 610041, Sichuan, China; ^2^ School of Basic Medical Sciences, Chengdu Medical College, Chengdu, 610041, Sichuan, China

**Keywords:** multidrug resistance, colorectal cancer, drug sensitivity, dishevelled, Wnt/β-catenin

## Abstract

Multidrug resistance is a great obstacle in successful chemotherapy of colorectal cancer. However, the molecular mechanism underlying multidrug resistance is not fully understood. Dishevelled, a pivot in Wnt signaling, has been linked to cancer progression, while its role in chemoresistance remains unclear. Here, we found that Dishevelled1-3 was over-expressed in multidrug-resistant colorectal cancer cells (HCT-8/VCR) compared to their parental cells. Silencing Dishevelled1-3 resensitized HCT-8/VCR cells to multiple drugs including vincristine, 5-fluorouracil and oxaliplatin. Moreover, Dishevelled1-3 increased the protein levels of multidrug resistance protein 1 (P-gp/MDR1), multidrug resistance-associated protein 2 (MRP2), and breast cancer resistance protein (BCRP), Survivin and Bcl-2 which are correlated with multidrug resistance. shβ-catenin abolished Dishevelled-mediated these protein expressions. Unexpectedly, none of Dishevelled1-3 controlled β-catenin accumulation and nuclear translocation. Furthermore, the nuclear translocations of Dishevelled1-3 were promoted in HCT-8/VCR cells compared to HCT-8. Dishevelled1-3 bound to β-catenin in nucleus, and promoted nuclear complex formation and transcription activity of β-catenin/TCF. Taken together, Dishevelled1-3 contributed to multidrug resistance in colorectal cancer via activating Wnt/β-catenin signaling and inducing the expressions of P-gp, MRP2, BCRP, Survivin and Bcl-2, independently of β-catenin accumulation and nuclear translocation. Silencing Dishevelled1-3 resensitized multidrug-resistant colorectal cancer cells, providing a novel therapeutic target for successful chemotherapy of colorectal cancer.

## INTRODUCTION

Colorectal cancer (CRC) is the third most prevalent malignancy and the fourth leading cause of cancer mortality worldwide [1]. Currently, surgery and chemotherapy are two major treatments for CRC. Systemic chemotherapy is often proposed as the first-line adjuvant treatment for patients with advanced CRC, aiming to palliate symptoms and prolong life [2]. Anticancer drugs such as vincristine, 5-fluorouracil and oxaliplatin are vital chemotherapy agents for CRC [3, 4]. However, the emergence of multidrug resistance (MDR) in CRC has greatly limited chemotherapeutic efficacy of the drugs, and finally results in therapy failure [5–7]. Therefore, overcoming MDR becomes a critical challenge in fighting against CRC.

The main mechanisms of MDR can be ranged into two principal types: “pump” and “non-pump” resistance [8, 9]. The major mechanism of pump resistance is involved in the increased ability of cancer cells to actively efflux drugs, which is induced by the ATP-binding cassette (ABC) superfamily of membrane transporters including ABCB1 (multidrug resistance protein 1, P-gp/MDR1), ABCC (multidrug resistance-associated protein, MRP) subfamily, and ABCG2 (breast cancer resistance protein, BCRP) [10]. These transporters over-expression can pump out several drugs and decrease intracellular drugs accumulation below toxic level, leading to an impairment of chemotherapeutic effects [11, 12]. On the other hand, the non-pump resistance is linked to apoptosis evasion which protects cancer cells from apoptosis [13]. The key proteins including Bcl-2 and Survivin inhibit multidrug-induced cell apoptosis forms and improve cancer cells survival, resulting in a decrease of chemotherapy sensitivity [14, 15]. Besides, increasing evidence has suggested that MDR is correlated with abnormal signaling transduction such as NF-κB, PI3K/AKT and Wnt signaling [9, 16]. In present study, we focused on the role of a critical regulator of Wnt signaling, disheveled (DVL), in MDR of CRC.

Wnt signaling plays an essential role in embryo development and adult tissue homeostasis, usually categorized as canonical and non-canonical signaling. The aberrant activation of canonical Wnt signaling (Wnt/β-catenin) triggers a wide variety of human pathologies such as various cancers [17]. The transcription factor complex, β-catenin/T-cell factor (TCF)/lymphoid enhancer factor (LEF), is regards as a major regulator of Wnt/β-catenin signaling. Without stimulation of Wnt ligand, β-catenin in cytoplasm is anchored by a destruction complex consisting of tumor suppressor adenomatous polyposis coli (APC), and glycogen synthase kinase-3 (GSK3β) and Axin. Subsequently, cytoplasmic β-catenin is phosphorylated by GSK3β and casein kinase 1(CK1), leading to ubiquitination and proteasomal degradation [18]. In the presence of available Wnt, it binds to the receptor Frizzled and co-receptor LDL receptor-related protein 5/6 on cell surface. This binding drives DVL to recruit Axin and GSK3β, thereby, allowing β-catenin to dissociate from the destruction complex. The phosphorylation and degradation of β-catenin is then inhibited, followed by β-catenin accumulation in cytoplasm and translocation into nucleus, where the binding of β-catenin to TCF/LEF induces expression of Wnt/β-catenin target gene [19]. Notably, independently of Wnt stimulation, over-expression of DVL can potently activate Wnt/β-catenin signaling [20]. A growing body of evidence suggests that DVL is correlated with tumor progression such as cancer cells proliferation, migration, invasion, tumorigenesis and metastasis [21, 22]. However, the effect of DVL on cancer chemoresistance remains poorly understood. Therefore, the role and underlying mechanism of DVL in MDR of CRC were investigated in this study.

## RESULTS

### Silencing DVL1-3 enhanced multidrug-induced cytotoxicity in CRC cells

**Figure 1:**
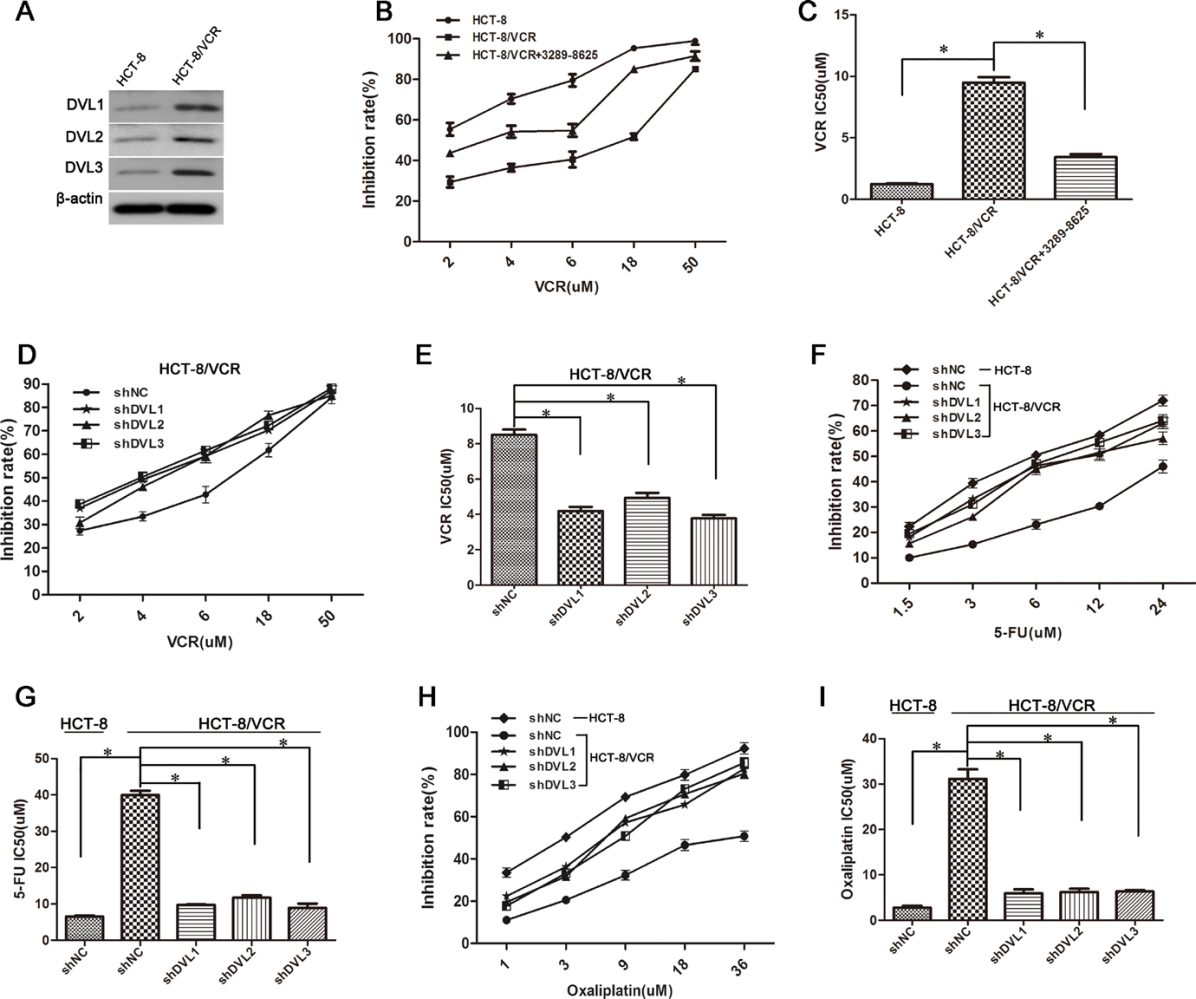
Silencing DVL promoted multidrug-induced cytotoxicity in CRC cells (**A**) HCT-8 and HCT-8/VCR cells were lysed to examine expressions of three DVL family members (DVL1-3). (**B**) The sensitivity of HCT-8, HCT-8/VCR and 3289-8625-treated HCT-8/VCR cells to vincristine was determined using MTT assays. (**C**) The IC50 of vincristine in HCT-8, HCT-8/VCR and 3289-8625-treated HCT-8/VCR cells. The sensitivity of HCT-8 and HCT-8/VCR cells transfected with shNC, shDVL1, shDVL2, or shDVL3, to vincristine (**D**), 5-FU (**F**) and oxaliplatin (**H**). The IC50 of vincristine (**E**), 5-FU (**G**) and oxaliplatin (I) in HCT-8 and HCT-8/VCR cells transfected with shNC, shDVL1, shDVL2, or shDVL3. Each experiment was performed in triplicate. ^*^,*P* < 0.05.

So far, there are very limited data about DVL and chemoresistance. Therefore, we evaluated the role of DVL in CRC resistance by analyzing the protein level of DVL in vincristine-resistant CRC cells HCT-8/VCR and their parental cells HCT-8. Data acquired showed that DVL1-3 was over-expressed in HCT-8/VCR cells compared with HCT-8, and HCT-8/VCR cells were more resistant to vincristine than HCT-8 cells (IC50 9.49 μM *vs* 1.23 μM) (Figure 1A–1C), suggesting that DVL was involved in CRC resistance to vincristine. We next explored the possibility that inhibiting DVL reverses the resistance in HCT-8/VCR cells to vincristine. In the presence of DVL inhibitor 3289–8625, the IC50 of vincristine in HCT-8/VCR cells was significantly reduced to 3.43 μM. Similar results were obtained in HCT-8/VCR cells transfected with shRNA targeting DVL. The sensitivity of HCT-8/VCR cells to vincristine was improved, and the IC50 of vincristine was significantly decreased by shDVL1 (4.19 μM), shDVL2 (4.94 μM) and shDVL3 (3.78 μM) compared with shNC (8.50 μM), respectively (Figure 1D and 1E). This suggested that silencing DVL1-3 enhanced vincristine-induced cytotoxicity in CRC cells.

Moreover, HCT-8/VCR cells exhibited more resistance to 5-FU (IC50 39.97 μM *vs* 6.53 μM) and oxaliplatin (31.15 μM *vs* 2.82 μM) compared with HCT-8 cells as well. However, silencing DVL using shDVL resensitized HCT-8/VCR cells to 5-FU and oxaliplatin. The IC50 of 5-FU (39.97 μM) in HCT-8/VCR cells was significantly decreased to 9.73, 11.72 and 8.90 μM by shDVL1, shDVL2 and shDVL3, respectively; and the IC50 of oxaliplatin (31.15 μM) was reduced to 5.96, 6.22 and 6.35 μM (Figure 1F–1I). Collectively, above data suggested that DVL was positively related to MDR in HCT-8/VCR cells, and silencing DVL1-3 can promote multidrug-induced cytotoxicity in CRC cells.

### DVL1-3 increased expression of ABC transporters

**Figure 2:**
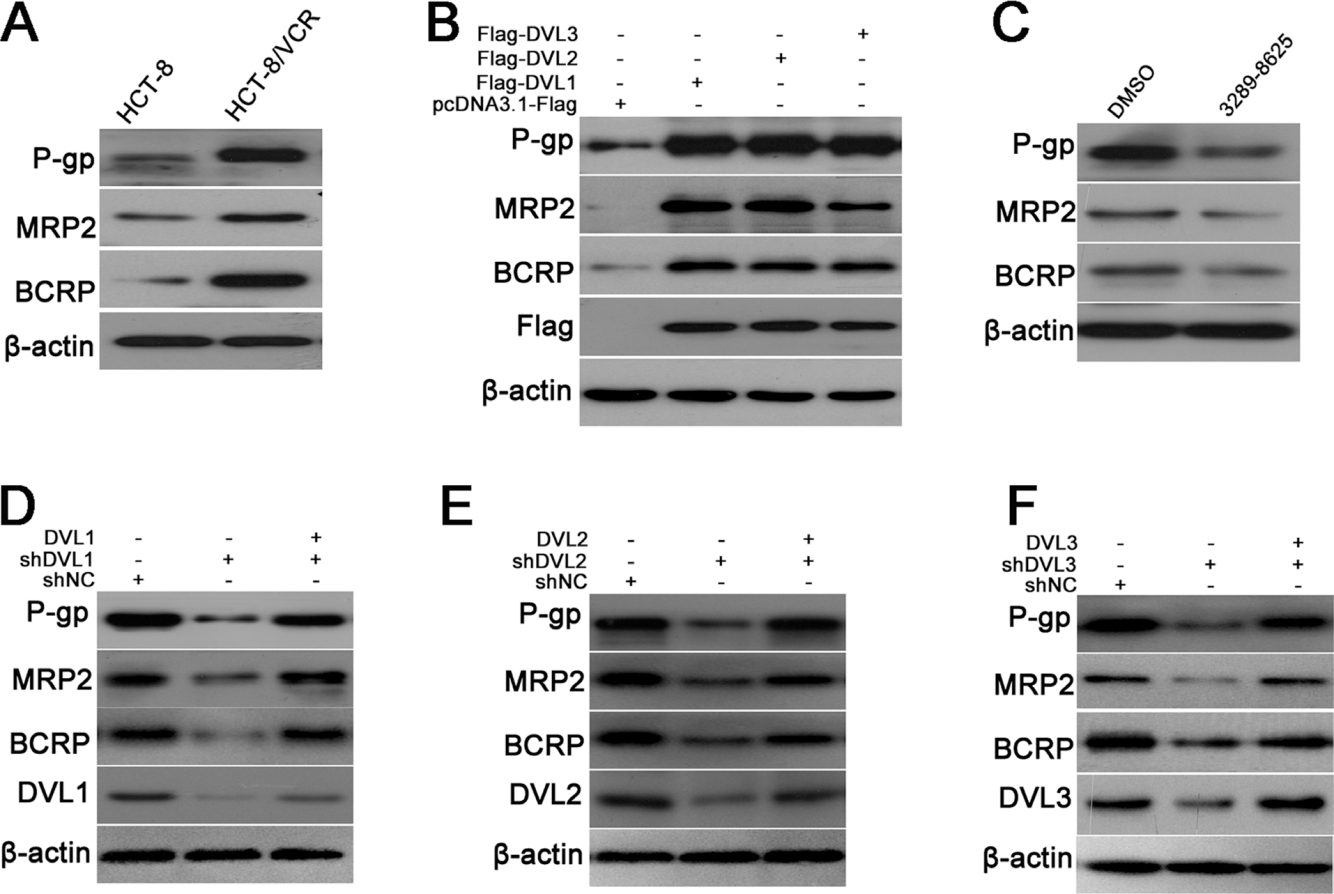
DVL increased expressions of P-gp, MRP2 and BCRP (**A**) Untreated HCT-8 and HCT-8/VCR cells, (**B**) HCT-8 cells transfected with pcDNA3.1-Flag-DVL or pcDNA3.1-Flag for 72 h, (**C**) HCT-8/VCR cells treated with or without 100 μM inhibitor 3289-8625 for 72 h, (**D**–**F**) HCT-8/VCR cells transfected with shNC, shDVL, or shDVL plus pcDNA3.1-Flag-DVL for 72 h, were lysed to examine the protein levels of P-gp, MRP2 and BCRP, Flag-DVL1-3 and DVL1-3 using Western blotting. In each case, the blot was representative of immunoblots resulting from three separate experiments.

To further explore the role of DVL in MDR of CRC, the expression of ABC transporters inducing MDR was examined in HCT-8/VCR and HCT-8 cells. The result showed that the protein levels of P-gp, MRP2 and BCRP were obviously increased in HCT-8/VCR cells compared to HCT-8 (Figure 2A). Given that DVL was also over-expressed in HCT-8/VCR cells, we measured whether DVL regulated the expressions of P-gp, MRP2 and BCRP. The pcDNA3.1-Flag recombinant vectors of DVL1, DVL2, and DVL3 were transfected into HCT-8 cells, respectively. Western blotting showed that the expressions of P-gp, MRP2 and BCRP were up-regulated by over-expressing DVL1, DVL2, and DVL3 compared to control group (Figure 2B).

To validate that the up-regulations of P-gp, MRP2 and BCRP were mediated by DVL, the compound 3289–8625 was used to inhibit DVL. As predicted, the expressions of P-gp, MRP2 and BCRP were down-regulated in HCT-8/VCR cells treated with 3289–8625 (Figure 2C). Similarly, the expressions of P-gp, MRP2 and BCRP were also decreased by shDVL1, shDVL2, and shDVL3. Moreover, shDVL and pcDNA3.1-DVL were co-transfected into HCT-8/VCR cells. shDVL1-, shDVL2- and shDVL3-induced decreases were respectively restored by over-expressions of DVL1, DVL2, and DVL3 (Figure 2D–2F). Taken together, above results suggested that DVL1-3 up-regulated the expressions of P-gp, MRP2 and BCRP to induce MDR in CRC.

### The effect of DVL1-3 on expression of anti-apoptosis protein and drugs-induced apoptosis

**Figure 3:**
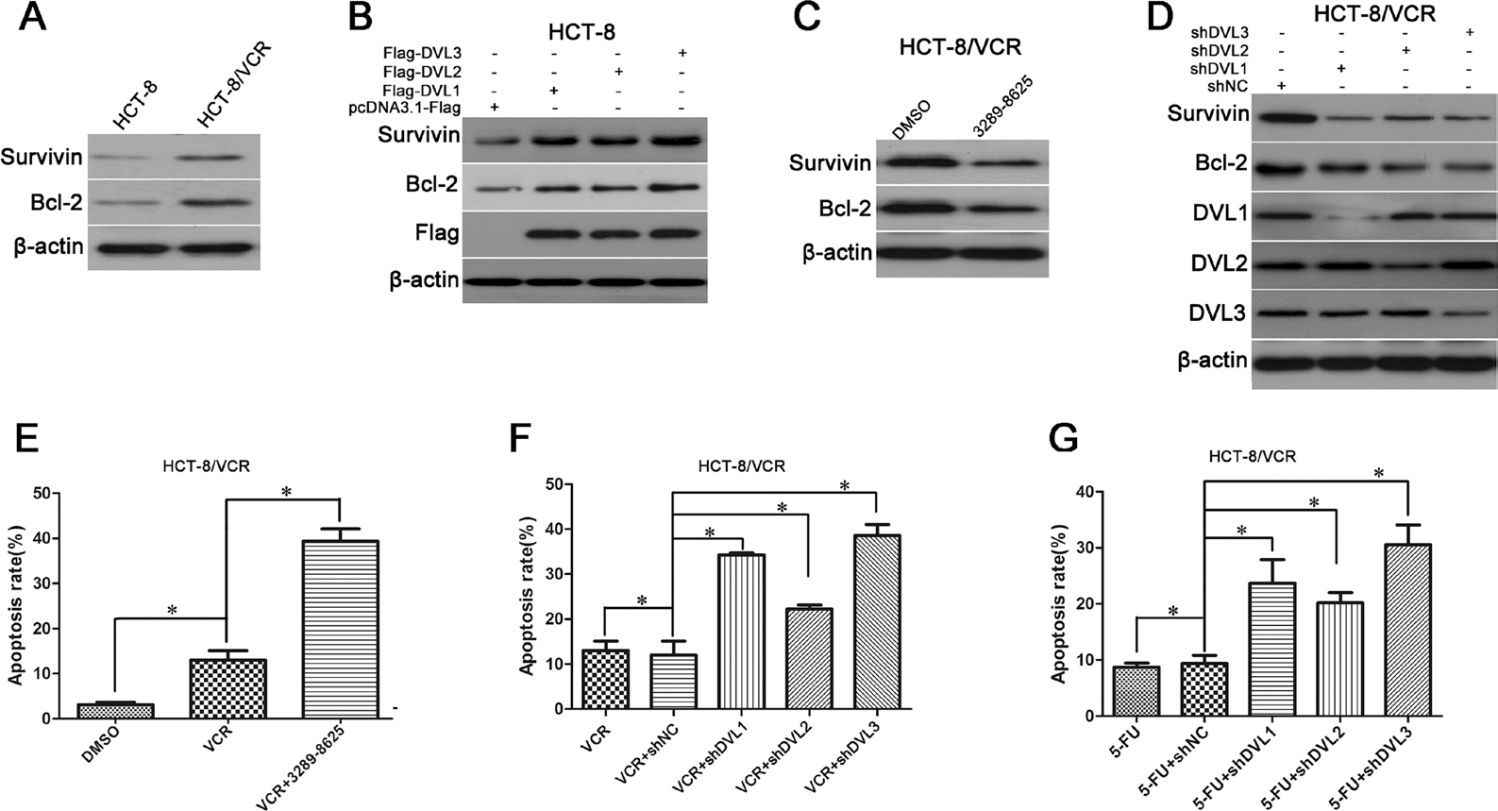
The effect of DVL on expressions of Survivin and Bcl-2, and drugs-induced apoptosis (**A**) Untreated HCT-8 and HCT-8/VCR cells, (**B**) HCT-8 cells transfected with pcDNA3.1-Flag-DVL or pcDNA3.1-Flag for 72 h, (**C**) HCT-8/VCR cells treated with or without 100 μM inhibitor 3289-8625 for 72 h, (**D**) HCT-8/VCR cells transfected with shNC or shDVL, for 72 h, were lysed to examine the protein levels of Survivin and Bcl-2, Flag-DVL1-3 and DVL1-3 using Western blotting. (**E**) HCT-8/VCR cells pretreated with or without 100 μM 3289-8625 for 24 h followed by treatment with 6 μM vincristine for additional 48 h, (**F**–**G**) HCT-8/VCR cells transfected with shNC or shDVL for 24 h followed by treatment with 6 μM vincristine and 12 μM 5-FU for additional 48 h, then flow cytometry experiment was used to analyze cell apoptosis. Each experiment was performed in triplicate. ^*^,*P* < 0.05.

Anti-apoptosis protein has also been shown to facilitate MDR [23]. Here, we analyzed expression difference of Survivin and Bcl-2 between HCT-8/VCR and HCT-8 cells. As shown in Figure 3A, Survivin and Bcl-2 were expressed at a higher level in HCT-8/VCR than HCT-8 cells. Next, we assessed whether DVL controlled the expressions of Survivin and Bcl-2. In addition, the protein levels of Survivin and Bcl-2 were increased in HCT-8 cells transfected with pcDNA3.1-Flag-DVL1, -DVL2, and -DVL3, while decreased in HCT-8/VCR cells treated with DVL inhibitor 3289–8625 (Figure 3B and 3C). The protein levels of Survivin and Bcl-2 were also down-regulated by shDVL1, shDVL2, and shDVL3 in HCT-8/VCR cells, (Figure 3D). The flow cytometry experiment showed that vincristine-induced apoptosis of HCT-8/VCR cells (13.03%) was promoted by 3289–8625 (39.36%) (Figure 3E). Furthermore, both vincristine- and 5-FU-induced apoptosis were also enhanced in HCT-8/VCR cells transfected with shDVL1 (34.27%; 23.68%), shDVL2 (22.21%; 20.19%), and shDVL3 (38.61%; 30.54%) relative to shNC (11.99%; 9.38%) (Figure 3F and 3G). These results suggested that DVL1-3 increased the expression of Survivin and Bcl-2 to suppress multidrug-induced apoptosis.

### β-catenin was responsible for DVL-mediated expression, while DVL1-3 did not regulate accumulation of β-catenin

**Figure 4:**
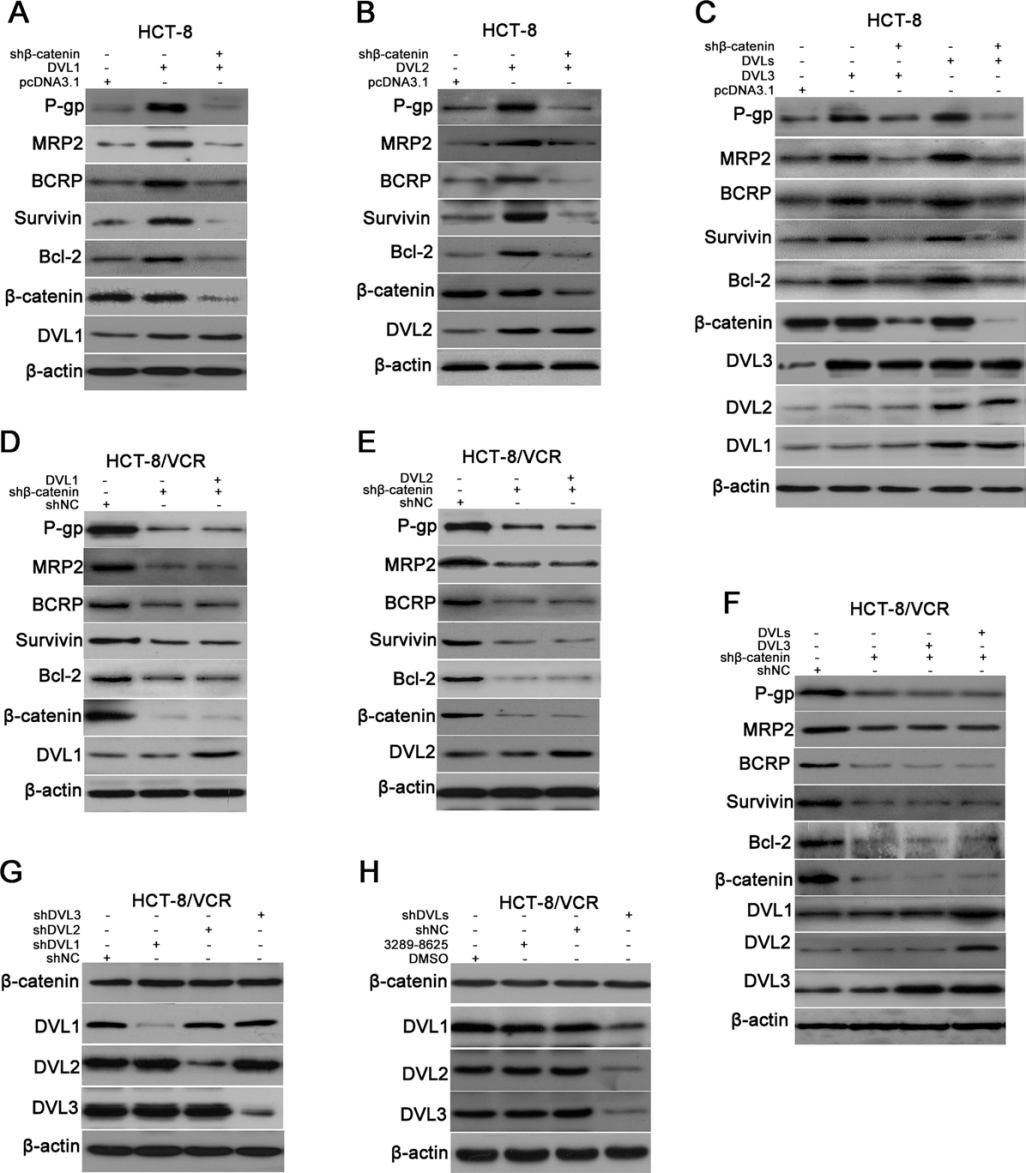
DVL up-regulated expressions of ABC transporters and anti-apoptosis genes via β-catenin, independently of β-catenin accumulation (**A**–**C**) HCT-8 cells transfected with pcDNA3.1-Flag, pcDNA3.1-Flag-DVL, cotransfected with pcDNA3.1-Flag-DVL and shβ-catenin, or cotransfected with pcDNA3.1-Flag-DVLs (pcDNA3.1-Flag-DVL1 plus pcDNA3.1-Flag-DVL2 and pcDNA3.1-Flag-DVL3) and shβ-catenin for 72 h, (**D**–**E**) HCT-8/VCR cells transfected with shNC, shβ-catenin, cotransfected with shβ-catenin and pcDNA3.1-Flag-DVL, or cotransfected with shβ-catenin and pcDNA3.1-Flag-DVLs for 72 h, (**G**) HCT-8/VCR cells transfected with shNC or shDVL for 72 h, (**H**) HCT-8/VCR cells treated with DMSO, 3289-8625, shNC, or shDVLs (shDVL1 plus shDVL2 and shDVL3) for 72 h, were lysed to examine expressions of P-gp, MRP2, BCRP, Survivin, Bcl-2, β-catenin and DVL1-3 using Western blotting, as indicated. In each case, the blot is representative of immunoblots resulting from three separate experiments.

Increasing evidence has suggested that several ABC transporters and anti-apoptosis genes are target genes of Wnt/β-catenin, including P-gp, MRP, BCRP, Survivin and Bcl-2 [24–28]. So we measured the role of β-catenin in DVL-mediated expression of P-gp, MRP2, BCRP, Survivin and Bcl-2. The recombinant vector of DVL was cotransfected with shβ-catenin into HCT-8 cells. The results showed that DVL1-, DVL2-, and DVL3-mediated expressions of P-gp, MRP2, BCRP, Surviving and Bcl-2 were eliminated by silencing β-catenin (Figure 4A–4C). Next, pcDNA3.1-Flag-DVL1 plus -DVL2 and -DVL3, named pcDNA3.1-DVLs, were cotransfected into HCT-8 cells to simultaneously over-express all three DVL family members, the effect of silencing β-catenin on DVLs-mediated expression was observed. The DVLs-induced up-regulations of P-gp, MRP2, BCRP, Survivin and Bcl-2 were also abolished by shβ-catenin in HCT-8 cells (Figure 4C), suggesting that β-catenin was required for DVL-mediated expression. As shown in Figure 4D–4F, it was sure that the expressions of P-gp, MRP2, BCRP, Survivin and Bcl-2 were decreased by shβ-catenin in HCT-8/VCR cells. However, none of over-expressions of DVL1-3 can restore expression of these proteins when β-catenin was silenced by shβ-catenin, even simultaneous over-expression of all three DVLs cannot up-regulated these protein levels. Above results suggested that DVL1-3 up-regulated expressions of P-gp, MRP2, BCRP, Survivin and Bcl-2 via β-catenin.

The accumulation of β-catenin is critical for Wnt/β-catenin target gene expression [29]. Therefore, we determined whether DVL controlled expression of β-catenin. Unexpectedly, neither respective over-expressions of DVL1-3 significantly changed the protein level of β-catenin in HCT-8 cells, nor simultaneous over-expression of DVLs up-regulated the accumulation of β-catenin (Figure 4A–4C). In addition, the down-regulation of β-catenin by shβ-catenin was not rescued by DVL1, DVL2, DVL3, or DVLs in HCT-8/VCR cells (Figure 4D–4F). Moreover, the expression of β-catenin was not significantly decreased by any one of shDVL1, shDVL2, and shDVL3 (Figure 4G), or DVL inhibitor 3289-8625 (Figure 4H). Subsequently, shRNA pools consisting of shDVL1 plus shDVL2 and shDVL3, named shDVLs, were cotransfected into HCT-8/VCR cells to synchronously silence all three DVLs. Similarly, we found that shDVLs cannot down-regulate the accumulation of β-catenin (Figure 4H). Collectively, these data indicated that β-catenin was required for DVL-mediated expressions of P-gp, MRP2, BCRP, Survivin and Bcl-2, while DVL1-3 did not regulate accumulation of β-catenin.

### The nuclear translocation of β-catenin was not controlled by DVL1-3

**Figure 5:**
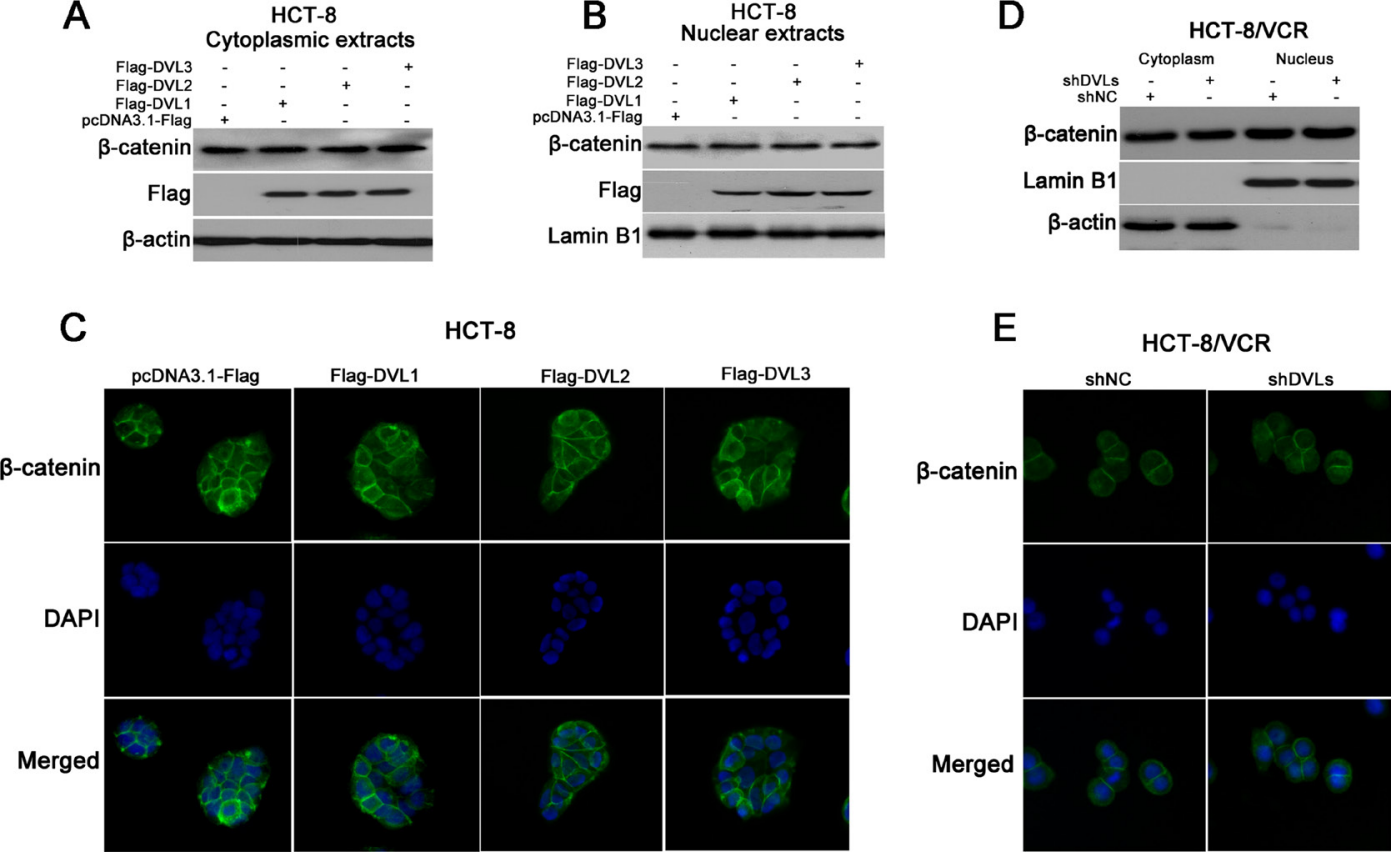
The effect of DVL on β-catenin nuclear translocation HCT-8 cells were transfected with pcDNA3.1-Flag or pcDNA3.1-Flag-DVL for 72 h, and then the protein levels of β-catenin and Flag-DVL1-3 in cytoplasmic (**A**) and nuclear (**B**) fractions were detected using Western blotting, the subcellular localization of β-catenin in HCT-8 cells was also analyzed using immunofluorescence staining (**C**). HCT-8/VCR cells were transfected with shNC or shDVLs for 72 h, the distribution of β-catenin between cytoplasm and nucleus were detected using Western blotting (**D**) and immunofluorescence staining (**E**). Green, β-catenin; blue, nuclear DNA.

It is well known that nuclear translocation of β-catenin contributes to Wnt/β-catenin target gene expression [30]. To understand how DVL promoted the expressions of P-gp, MRP2, BCRP, Survivin and Bcl-2, we examined the effect of DVL on the subcellular localization of β-catenin in HCT-8 and HCT-8/VCR cells. Western blotting showed that the protein levels of both cytoplasmic and nuclear β-catenin were not remarkably changed in HCT-8 cells transfected with recombinant vector of DVL (Figure 5A and 5B). Similarly, the immunofluorescence staining showed that none of over-expressions of DVL1, DVL2, and DVL3 triggered nuclear translocation of β-catenin in HCT-8 cells (Figure 5C). On the other hand, western blotting revealed that synchronously silencing all three DVLs using shDVLs still did not significantly disturb the distribution of β-catenin between cytoplasm and nucleus in HCT-8/VCR cells (Figure 5D). This was confirmed by the result of immunofluorescence staining that shDVLs did not reduce the nuclear translocation of β-catenin (Figure 5E). Above results suggested that the nuclear translocation of β-catenin is not controlled by DVL1-3.

### DVL1-3 enhanced nuclear complex formation of β-catenin/TCF and its transcription activity

**Figure 6:**
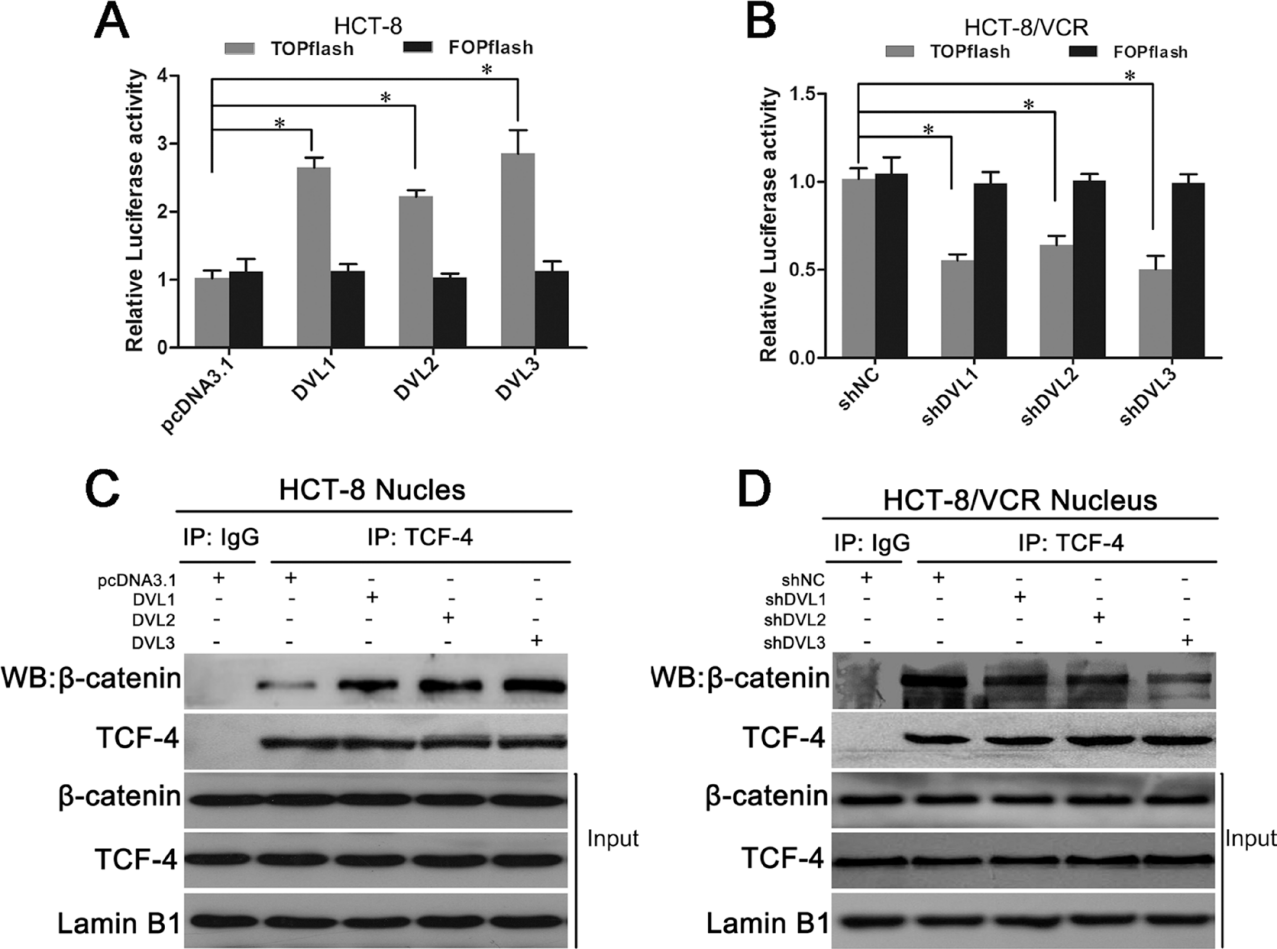
DVL promoted nuclear complex formation and transcription activity of β-catenin/TCF (**A**) In HCT-8 cells, pcDNA3.1-Flag or pcDNA3.1-Flag-DVL was cotransfected with 0.2 μg of TOPflash plus10 ng of pRL-SV40 or FOPflash plus10 ng of pRL-SV40; (**B**) in HCT-8/VCR cells, shNC or shDVL was cotransfected with 0.2 μg of TOPflash plus10 ng of pRL-SV40 or FOPflash plus10 ng of pRL-SV40. After transfection for 48 h, TOPflash and FOPflash luciferase activity were determined using a dual-luciferase reporter assay system. FOPflash was used as a specificity control for TOPflash activity. The relative luciferase activity was measured by normalization to Renilla reporter pRL-SV40 activity. (**C**) HCT-8 cells transfected with pcDNA3.1-Flag or pcDNA3.1-Flag-DVL for 48 h, (**D**) HCT-8/VCR cells transfected with shNC or shDVL for 48 h, were lysed to extract nuclear fractions. The nuclear fractions were incubated with anti-TCF-4 antibody for IP experiment. IgG was used as negative control. The immunoprecipitates and nuclear fractions were subjected to Western blotting using anti-β-catenin and anti-TCF-4 antibodies. Each experiment was performed in triplicate. ^*^P < 0.05.

Next, we evaluated that the effect of DVL on nuclear β-catenin activity. TOPflash and FOPflash luciferase reporters, which respectively contain the wildtype and mutated β-catenin/TCF-binding site, were extensively used to characterize β-catenin/TCF-dependent transcription activity in nucleus [31]. TOPflash or FOPflash reporter plasmid was cotransfected with pcDNA3.1-Flag-DVL into CRC cells. Dual-luciferase reporter assay showed that TOPflash luciferase activity was higher in HCT-8 cells transfected with pcDNA3.1-Flag-DVL1 (2.58 fold), -DVL2 (2.16 fold), or -DVL3 (2.78 fold) relative to control group (Figure 6A), while lower in HCT-8/VCR cells transfected with shDVL1 (45.57% reduction), shDVL2 (37.05 % reduction), or shDVL3 (50.49% reduction) relative to shNC (Figure 6B). However, there was no significant difference in FOPflash luciferase activity after over-expression and silence of DVL1-3 compared to the control (Figure 6A and 6B). These data suggested that DVL1-3 specifically enhanced β-catenin/TCF-dependent transcription activity.

To gain insights into the mechanism underlying that DVL up-regulated the β-catenin/TCF-dependent transcription activity, we detected effect of DVL on the nuclear complex formation of β-catenin/TCF. Co-immunoprecipitation (Co-IP) assay showed that the binding of β-catenin to TCF-4 was increased in nucleus of HCT-8 cells transfected with pcDNA3.1-DVL1, -DVL2, or -DVL3 (Figure 6C), while decreased in nucleus of HCT-8/VCR cells transfected with shDVL1, shDVL2, or shDVL3 (Figure 6D). However, the total protein levels of nuclear β-catenin and TCF-4 were not significantly changed by over-expression and silence of DVL1-3 (Figure 6C and 6D). This suggested that DVL1-3, independently of nuclear accumulation of β-catenin and TCF-4, enhanced the nuclear complex formation or stability of β-catenin/TCF to increase β-catenin- driven transcription activity.

### DVL1-3 bound to β-catenin in nucleus

**Figure 7:**
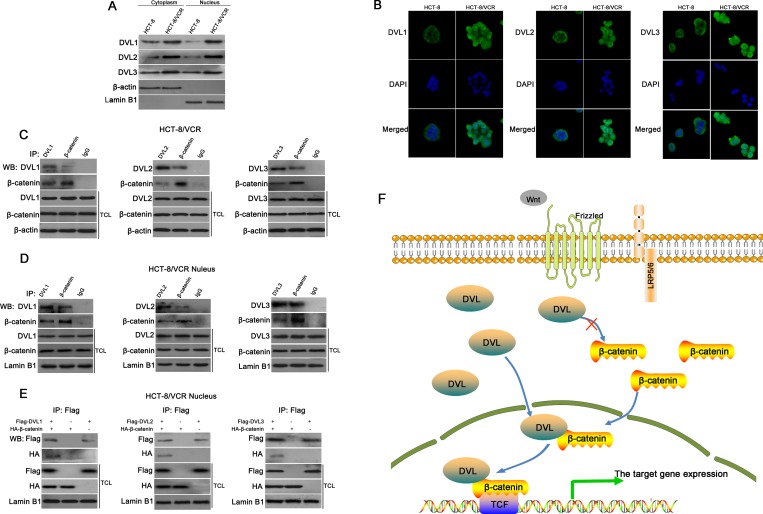
DVL bound to β-catenin in nucleus The distributions of DVL between cytoplasm and nucleus were measured using Western blotting (**A**) and immunofluorescence staining (**B**) in HCT-8 and HCT-8/VCR cells. HCT-8/VCR cells transfected without (**C**–**D**) or with (**E**) pcDNA3.1-Flag-DVL and pcDNA3.1-HA-β-catenin for 72 h, were lysed to extract total cellular or nuclear fractions. The fractions were incubated with anti-DVL1, anti-DVL2, anti-DVL3, anti-β-catenin, or anti-Flag antibody for IP experiment. IgG was used as negative control. The immunoprecipitates and fractions were subjected to Western blotting using antibody as indicated. In each case, the blot was representative of immunoblots resulting from three separate experiments. (**F**) A model for involvement of DVL as a coactivator in Wnt/β-catenin signaling.

To further understand how DVL stabilized nuclear β-catenin/TCF complex, we checked the nuclear translocation of DVL in HCT-8/VCR and HCT-8 cells. Here, we found that the protein levels of DVL1, DVL2 and DVL3 in cytoplasm and nucleus of HCT-8/VCR cells were higher than HCT-8 cells (Figure 7A). Immunofluorescence staining also showed that DVL1, DVL2 and DVL3 were more abundantly localized in the nucleus of HCT-8/VCR cells (Figure 7B). Moreover, the result of co-immunoprecipitation (Co-IP) revealed that endogenous nuclear DVL1-3 and β-catenin were precipitated down by each other in HCT-8/VCR cells (Figure 7C and 7D). Furthermore, the recombinant vector of Flag-DVL and HA-β-catenin were transfected into HCT-8/VCR cells. The fusion proteins Flag-DVL1-3 were physically associated with HA-β-catenin in the nucleus (Figure 7E). These data suggested that nuclear DVL1-3 bound to β-catenin and may contribute to the complex formation of β-catenin/TCF.

## DISCUSSION

CRC is a major cause of cancer-related death throughout the world. Significant improvements in survival rates of CRC patient have been achieved in recent years due to advances in cancer treatment. However, the resistance to chemotherapy agents usually occurs during advanced CRC treatment and facilitates cancer progression [32, 33]. The most common and standard strategy against resistance is to employ other anti-cancer drugs, but this does not always work in most cases because of MDR [34]. The development of MDR is considered as a major impediment in CRC treatment [6]. Thus, it is urgent to understand the mechanisms responsible for MDR in CRC and develop efficient therapeutic strategies to overcome chemoresistance. Our present study showed that DVL family members contributed to MDR in CRC. DVL1-3 bound to β-catenin in nucleus, acted as a coactivator to promote the complex formation of β-catenin/TCF and its transcription activity, triggering the expression of P-gp, BCRP, MRP2, Survivin and Bcl-2 which are correlated with MDR and the target genes of Wnt/β-catenin, independently of β-catenin accumulation and nuclear translocation. Silencing DVL restored the sensitivity of CRC cells to multiple drugs, suggesting that DVL can be considered as a molecular target for CRC chemotherapy.

Although earlier studies have shown that DVL is relevant to tumor progression [35, 36], its role in chemoresistance remains poorly understood. Here, we found all three DVL family members (DVL1-3) were expressed at a higher level in vincristine-resistant CRC cells HCT-8/VCR compared to their parental cells HCT-8 (Figure 1A–1C), indicating that over-expression of DVL was involved in resistance of CRC to vincristine. To evaluate the effect of DVL on vincristine-resistance, the inhibitor 3289-8625, a cell-permeable aminobenzanilide compound, was used to pharmacologically suppress the function of DVL. Although HCT-8/VCR cells exhibited much stronger resistance to vincristine compared to HCT-8, the DVL inhibitor 3289–8625 resensitized HCT-8/VCR cells to vincristine, and decreased the IC50 value (Figure 1B and 1C). We further evaluated the role of DVL in MDR. Cancer cells which obtain resistance to one drug are universally resistant to other structurally and functionally irrelevant cytotoxic agents as well [37]. Expectedly, HCT-8/VCR cells were cross-resistant to 5-FU and oxaliplatin, suggesting that they can be used as a MDR model. Next, the expressions of DVL1-3 were silenced by corresponding shRNA. shDVL1-3 promoted sensitivity of HCT-8/VCR cells to vincristine, 5-FU and oxaliplatin, and markedly reduced the IC50 values of these drugs (Figure 1D–1I). Above results suggested that DVL1-3 positively controlled MDR in CRC, and down-regulating DVL chemosensitized drugs-induced cytotoxicity in multidrug-resistant CRC cells.

Next, we focused on the underlying mechanism that DVL regulated MDR in CRC. One of the major mechanisms of MDR is closely linked to ABC membrane transporters which can pump multiple chemotherapy agents out of cells to attenuate drugs-induced cytotoxicity, such as P-gp, BCRP and MRP [38, 39]. Here, we found that P-gp, BCRP and MRP2 were over-expressed in multidrug-resistant CRC cells HCT-8/VCR (Figure 2A), suggesting that these proteins facilitated MDR in CRC. Moreover, the expressions of P-gp, BCRP and MRP2 were increased by up-regulation of DVL1-3 in HCT-8 cells (Figure 2B), but decreased by down-regulation of DVL1-3 using compound 3289–8625 or shDVL1-3 in HCT-8/VCR cells. Meanwhile, these decreases mediated by shDVL1-3 were respectively rescued by over-expression of DVL1-3 (Figure 2C–2F). These data indicated that DVL positively controlled the expression of P-gp, BCRP and MRP2 to facilitate MDR in CRC. The emergence of MDR is not limited to ectopic expression of ABC transporters. Besides, anti-apoptosis proteins also contribute to MDR [40]. The over-expressions of Survivin and Bcl-2 are often found in a series of solid tumors including CRC. The sustained high-level expression of anti-apoptosis protein protects CRC cells from drugs-induced apoptosis to mediate MDR [41, 42]. The current study showed that Survivin and Bcl-2 were over-expressed in HCT-8/VCR cells (Figure 3A). DVL1-3 positively regulated the protein levels of Survivin and Bcl-2 (Figure 3B–3D). Furthermore, silencing DVL1-3 promoted vincristine- and 5-FU-induced apoptosis (Figure 3E and 3G). Taken together, above data suggested that DVL1-3 not only increased the expressions of ABC transporters in favor of drugs efflux, but also up-regulated anti-apoptosis proteins against drugs-induced apoptosis, finally leading to MDR of CRC.

Previous studies have certified that P-gp, BCRP, MRP, Survivin and Bcl-2 are target genes of Wnt/β-catenin signaling [24–28]. Therefore, we determined the role of β-catenin in the DVL-induced expression of these ABC transporters and anti-apoptosis proteins in CRC. The present results revealed that silencing β-catenin abolished the up-regulation of P-gp, BCRP, MRP2, Survivin and Bcl-2 mediated by DVL1-3 in HCT-8 cells (Figure 4A–4C). Meanwhile, DVL1-3 lost ability to up-regulate P-gp, BCRP, MRP2, Survivin and Bcl-2 when β-catenin was silenced in HCT-8/VCR cells (Figure 4D–4F). These results suggested that DVL1-3 up-regulated the expression of P-gp, BCRP, MRP2, Survivin and Bcl-2 via β-catenin. The accumulation of β-catenin in cells is an important step to trigger Wnt/β-catenin target gene expression [43]. Unexpectedly, neither DVL1-3 over-expression nor silence can significantly change the protein level of β-catenin (Figure 4), suggesting that DVL1-3 mediated the expression of P-gp, BCRP, MRP2, Survivin and Bcl-2, independently of β-catenin accumulation. It is known that nuclear translocation of β-catenin following the accumulation is another critical step to induce Wnt/β-catenin target gene expression [44]. So we hypothesized that although DVL did not affect β-catenin accumulation, it may drive β-catenin nuclear translocation to induce the expression of P-gp, BCRP, MRP2, Survivin and Bcl-2. However, this hypothesis was not supported by our results that none of DVL1-3 can significantly regulate the distribution of β-catenin between cytoplasm and nucleus in HCT-8 and HCT-8/VCR cells (Figure 5). These data suggested that β-catenin was required for DVL-mediated the expression of P-gp, BCRP, MRP2, Survivin and Bcl-2 in CRC cells, while DVL1-3 neither controlled the accumulation nor nuclear translocation of β-catenin.

It has been shown that DVL can increase the accumulation of β-catenin through inactivation of destruction complex containing APC, GSK3β and Axin, leading to translocation of β-catenin into nucleus [45, 46]. However, the functional destruction complex has been usually disrupted in APC-mutant CRC cells including HCT-8 cells [47–50], and our present data showed that although DVLs suppressed GSK3β activity in HCT-8 cells, the inactivation of GSK3β did not change the protein level of β-catenin in cytoplasm and nucleus (Supplementary Figure 1). This may explain why DVL did not regulate the accumulation and nuclear translocation of β-catenin in HCT-8 and its resistant cells HCT-8/VCR. Growing evidence suggests that β-catenin is tightly regulated at three hierarchical levels: protein accumulation, subcellular localization and transcription activity [51, 52]. Given that DVL triggered the expression of P-gp, BCRP, MRP2, Survivin and Bcl-2 known as Wnt/β-catenin target genes via β-catenin, independently of the accumulation and nuclear translocation of β-catenin, we reasoned that DVL may function in transcription activity of β-catenin to trigger the Wnt/β-catenin target genes expression in CRC cells carrying APC mutant. Next, we examined whether DVL regulated β-catenin-driven transcription using TOPflash or FOPflash reporter plasmid in HCT-8 and HCT-8/VCR cells. Here, we found that FOPflash luciferase activity was positively controlled by DVL1-3, while the FOPflash luciferase activity was not significantly affected (Figure 6A and 6B), suggesting that DVL1-3 specifically activated β-catenin/TCF-dependent transcription. The binding of β-catenin to TCF in nucleus is critical for β-catenin/TCF-dependent transcription activity [53]. Our result showed that the physical association of nuclear β-catenin with TCF-4 was up-regulated by DVL1-3 over-expression in HCT-8 cells, while down-regulated by shDVL1-3 in HCT-8/VCR cells. However, the total protein levels of nuclear β-catenin and TCF-4 were not significantly changed by DVL1-3 (Figure 6C and 6D). Collectively, above data suggested that DVL enhanced the complex formation of β-catenin/TCF, independently of nuclear β-catenin and TCF-4 accumulation, to activate β-catenin/TCF-dependent transcription and Wnt/β-catenin target genes expression.

Previous reports have shown that DVL can increase β-catenin accumulation in cytoplasm to enhance translocation of β-catenin into nucleus, consequently promote nuclear complex formation of β-catenin/TCF and Wnt/β-catenin signaling [54, 55]. However, the current study showed that DVL neither changed the accumulation of β-catenin, nor drove its nuclear translocation. Therefore, we further explored the mechanism underlying that DVL promoted the complex formation of β-catenin/TCF. DVL is a pivot bridging the receptors and downstream components of Wnt signaling, and can shuttle between cytoplasm and nucleus [56]. The nuclear translocation of DVL has shown to be required for its role in Wnt/β-catenin [56, 57]. In this study, we found that all three DVL family members were more abundantly localized in the nucleus of HCT-8/VCR cells compared to HCT-8 (Figure 7A and 7B), suggesting that the nuclear translocations of DVL1-3 were involved in MDR of CRC, and provided facilities for enhancing the nuclear complex formation of β-catenin/TCF. Earlier studies have indicated that the binding of β-catenin to TCF is not sufficient, albeit necessary, for target gene activation of Wnt/β-catenin which requires binding of coactivators, such as BCl9, CBP and Pygopus, to β-catenin in nucleus [58]. Our results showed that endogenous nuclear DVL1-3 were coimmunoprecipitated with β-catenin, and Flag-DVL1-3 proteins were also physically associated with HA-β-catenin in nucleus (Figure 7C–7E), suggesting that DVL1-3 bound to β-catenin, may function as a coactivator to facilitate the complex formation of β-catenin/TCF and its transcription activity. This was consistent with previous report showing that binding of DVL to β-catenin stabilized β-catenin/TCF complex to promote its transcription activity [59].

In conclusion, this study revealed that DVL contributed to MDR in CRC. All three DVL family members were over-expressed in multidrug-resistant CRC cells, where their nuclear translocations were increased. DVL1-3 bound to β-catenin in nucleus, acted as a coactivator to enhance the complex formation of β-catenin/TCF and its transcription activity (Figure 7F), inducing the expression of ABC transporters and anti-apoptosis genes correlated with MDR, independently of β-catenin accumulation and nuclear translocation. Silencing DVL resensitized multidrug-resistant CRC cells, providing a new target for overcoming MDR in CRC.

## MATERIALS AND METHODS

### Materials

The primary antibodies used in the present study: anti-DVL1, anti-DVL2, anti-DVL3, anti-β-catenin, anti-Flag, anti-HA, anti-Lamin B1 and anti-β-actin were obtained from Santa Cruz Biotechnology (Santa Cruz, USA); anti-TCF-4, anti-P-gp, anti-MRP2, anti-BCRP, anti-GSK3β, anti-p-GSK3β (Ser 9), anti-Survivin and anti-Bcl-2 were purchased from Cell Signaling Technology (Danvers, USA). The secondary antibodies were obtained from ZSGB-bio (Peking, China). shDVL1 (target sequence: 5′-GGAGGAGATCTTTGATGAC-3′), shDVL2 (target sequence, 5′-GGAAGAAATTTCAGATGAC-3′), shDVL3 (target sequence: 5′-GGAGGAGATCTCGGATGAC-3′) and shNC (negative control) were purchased from Genechem (Shanghai, China). cDNA encoding DVL1 was cloned into pcDNA3.1-Flag to construct DVL1 recombinant vector pcDNA3.1-Flag-DVL1. pcDNA3.1-HA-β-catenin was constructed in a similar manner. pcDNA3.1-Flag-DVL2 and pcDNA3.1-Flag-DVL3 were derived from pCMV5-3XFlag-DVL2 and XE251 pCDNA3.1(zeo)-hDsh3 which are gifts from Jeff Wrana (Addgene plasmid # 24802) [60] and Randall Moon (Addgene plasmid # 16758) [61], respectively. The culture medium RPMI-1640, fetal bovine serum (FBS), penicillin, and streptomycin were purchased from HyClone Laboratories (Logan, USA). Lipofectamine 3000 transfection reagent and Annexin V-FITC/PI apoptosis detection kit were purchased from Invitrogen (Carlsbad, USA). RIPA (Radioimmunoprecipitation) lysis buffer, protein A+G agarose beads and BCA protein assay kit were obtained from Beyotime Biotechnology (Nantong, China).

### Cell culture

The human colorectal cancer cell line HCT-8 and its vincristine-resistant subline HCT-8/VCR were obtained from Shanghai Bogoo Biotechnology (Shanghai, China). The cells were authenticated by STR profile, and cultured in RPMI-1640 medium supplemented with 10% fetal bovine serum, 100 μg/ml streptomycin and 100 units/ml penicillin at 37°C in a humidified 5% CO2 atmosphere. 0.5 μM vincristine sulfate was added into the medium to maintain drug-resistance phenotype of HCT-8/VCR cells.

### Drug cytotoxicity

Drug cytotoxicity was evaluated using MTT assay. Briefly, cells (4 × 10^3^/well) were seeded into each well of a 96-well plate. After incubation for 24 h, cells were treated with 100 μM DVL inhibitor 3289–8625 (Merck, Darmstadt, Germany) or transfected with shDVL for 24 h, followed by treatment with a range of concentrations of vincristine, 5-FU or oxaliplatin (Sigma-Aldrich, St Louis, USA), for another 48 h. The cells were then washed twice using PBS, and 100 μl of 0.5 mg/ml MTT in medium culture was added to each well for 4 h incubation at 37°C. Following the culture medium was discarded, 150 μl DMSO was added to dissolve formazan blue. Absorbance at 490 nm was measured using an Ultra Microplate Reader (Bio-Tek Instruments, Winooski, USA). The inhibition ratio and IC50 (drug concentrations that achieved 50% growth inhibition) were calculated from the survival curves using the Bliss method.

### Cell transfection and cotransfection

Cells were cell dissociated using a 0.25% trypsin solution when they reached to approximately 90% confluence, and cultured in 96- 24- or 6-well plates until 70–90% confluent. Next, the DNA recombinant vectors and shRNA were respectively transfected or cotransfected into cells using Lipofectamine 3000 transfection reagent according to the manufacturer's instructions, and the cells were incubated at 37°C under a humidified 5% CO2 atmosphere for 72 h before harvest.

### Cell apoptosis

The extent of apoptosis was measured through Annexin V-FITC/PI apoptosis detection kit according to the manufacturer's instruction. Briefly, cells were seed in 24-well plates and pretreated with 3289–8625 or transfected with shDVL for 24 h, followed by treatment with vincristine (6 μM) or 5-FU (12 μM) for additional 48 h. The cells were washed with cold PBS twice, gently resuspended in 100 μl binding buffer. The cell suspension was incubated with 1.5 μl Annexin V-FITC for 10 min at 4°C in the dark, and then 2.5 μl PI for 5 min. Apoptotic cells were quantified using a flow cytometer (BD FACSCalibur, San Jose, USA) and the CellQuest software. At least 10,000 cells were analyzed for each sample.

### Western blotting analysis

Total cell proteins were obtained using RIPA lysis buffer. The cytoplasmic and nuclear proteins were extracted as described previously [62]. Protein concentration was assessed using a BCA protein assay kit. The equal amount of proteins (100 μg/sample) were loaded into each lane of sodium dodecyl sulfate–polyacrylamide gel (10%) and electrophoresed. The resolved proteins were electrophoretically transferred to PVDF membranes. Following blocking with 5% non-fat milk at room temperature for 2 h, the membranes were incubated with primary antibodies at 4°C overnight, and then with HRP-conjugated secondary antibodies for 2 h at room temperature. Specific immune complexes were detected using chemiluminescence reagent plus Bio-Rad Molecular Imager.

### Immunofluorescence assay

Cells were cultured on chamber slides to be 40–60% confluence, and then washed three times with phosphate buffer solution (PBS), fixed with 4% paraformaldehyde for 20 min, permeabilized using 0.2% Triton X-100 for 20 min, and blocked with 10% goat serum for 2 h at room temperature. Cells were then incubated with antibody against β-catenin or DVL (1:50 dilution) overnight at 4°C. The slides were washed three times with PBS, followed by incubation with Alexa Fluor 488-conjugated secondary antibodies (1:1000 dilution) for 2 h at room temperature. Nucleus was stained with DAPI (5 μg/ml) for 10 min and washed with PBS twice. The cells were visualized under a Zeiss confocal laser scanning microscope.

### Luciferase activity assay

TOPflash and FOPflash constructs (Upstate Biotechnology, Lake Placid, USA) are usually employed to assess β-catenin/TCF-dependent transcription [31]. TOPflash construct driven by thymidine kinase promoter contains SIX wildtype β-catenin/TCF-binding sites upstream of a luciferase reporter gene. FOPflash is similar as TOPflash except that it contains SIX mutated β-catenin/TCF-binding sites. FOPflash is employed as a specific control for TOPflash activity. Cells were seed in 24-well plates at 4 × 10^4^ cells per well and cultured to approximately 70% confluency. Then, pcDNA3.1-Flag-DVL and shDVL were respectively cotransfected with 0.2 μg of TOPflash plus 10 ng of pRL-SV40 or FOPflash plus 10 ng of pRL-SV40 by using Lipofectamine 3000 transfection reagent. After 48 h, the TOPflash and FOPflash luciferase activity were determined using a dual-luciferase reporter assay system (Promega Corporation, Madison, USA). The luciferase activity of each sample was normalized against Renilla reporter pRL-SV40 (Promega Corporation, Madison, USA) luciferase activity for monitoring transfection efficiency.

### Coimmunoprecipitation

Briefly, the protein extracts were incubated with 2 μg of anti-TCF-4, anti-β-catenin or anti-DVL antibody at 4°C overnight. Thereafter, protein A+G agarose beads were added into the mixture and incubated at 4°C for 6 h. The complexes were collected by centrifuging at 3,000 × g for 5 min at 4°C, and the precipitates were washed four times with ice-cold PBS. Bound proteins were separated from the agarose beads by boiling in sample buffer for 10 min, followed by western blotting analysis. The fusion proteins Flag-DVL and HA-β-catenin were immunoprecipitated and detected by incubation with anti-Flag and anti-HA antibody.

### Statistical analysis

Results were reported as mean ± standard deviation (SD) of three independent experiments unless otherwise specified. Data were analyzed by two-tailed unpaired Student's *t*-test between two groups and by one-way ANOVA followed by Bonferroni test for multiple comparison involved. These analyses were performed using GraphPad Prism Software Version 5.0 (GraphPad Software Inc, La Jolla, USA), level of significance was set at a *P* < 0.05.

## SUPPLEMENTARY MATERIALS FIGURE


