# Correction: Dishevelled1-3 contribute to multidrug resistance in colorectal cancer via activating Wnt/β-catenin signaling

**DOI:** 10.18632/oncotarget.28142

**Published:** 2021-11-23

**Authors:** Kun Zhang, Minhui Li, Houyi Huang, Linpeng Li, Jie Yang, Li Feng, Junjie Gou, Mengju Jiang, Liaotian Peng, Linyi Chen, Ting Li, Ping Yang, Yuhan Yang, Yuanyuan Wang, Quekun Peng, Xiaozhen Dai, Tao Zhang

**Affiliations:** ^1^ School of Biomedical Sciences, Chengdu Medical College, Chengdu, 610041, Sichuan, China; ^2^ School of Basic Medical Sciences, Chengdu Medical College, Chengdu, 610041, Sichuan, China; ^*^ These authors contributed equally to this work

**This article has been corrected:** In Figure 2E, the MRP2 blot panel was inadvertently duplicated in the BCRP blot panel. The corrected Figure 2E, produced using the original data, is shown below. The authors declare that these corrections do not change the results or conclusions of this paper.

Original article: Oncotarget. 2017; 8:115803–115816. 115803-115816. https://doi.org/10.18632/oncotarget.23253

**Figure 2:**
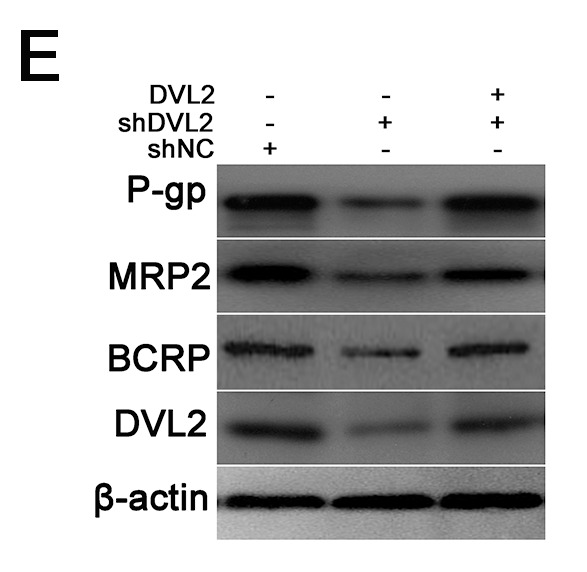
DVL increased expressions of P-gp, MRP2 and BCRP. (**E**) HCT-8/VCR cells transfected with shNC, shDVL, or shDVL plus pcDNA3.1-Flag-DVL for 72 h, were lysed to examine the protein levels of P-gp, MRP2 and BCRP, Flag-DVL1-3 and DVL1-3 using Western blotting. In each case, the blot was representative of immunoblots resulting from three separate experiments.

