# Supplementary material for: Dishevelled1-3 contribute to multidrug resistance in colorectal cancer via activating Wnt/β-catenin signaling
Source: Oncotarget. 2017 Dec 14;8(70):115803–16. doi: 10.18632/oncotarget.23253 (PMC5777814; doi:10.18632/oncotarget.23253)
Supplement: Supplementary file 1 [file oncotarget-08-115803-s001.pdf]

Dishevelled1-3 contribute to multidrug resistance in colorectal cancer via activating Wnt/β-catenin signaling

SUPPLEMENTARY MATERIALS

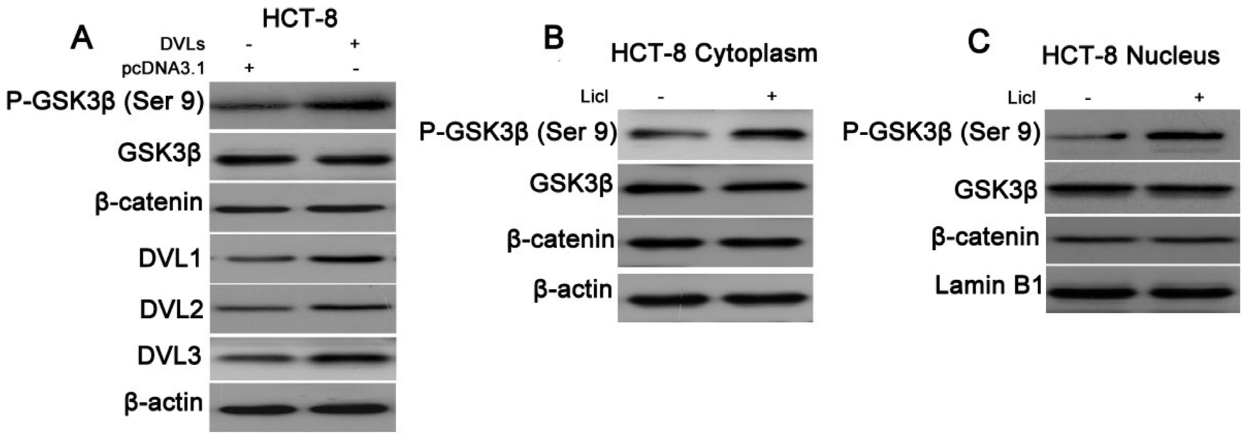

Supplementary Figure 1: (**A**) DVL inhibited GSK3β activity. HCT-8 cells transfected with pcDNA3.1-Flag, or transfected with pcDNA3.1-Flag-DVLs (pcDNA3.1-Flag-DVL1 plus pcDNA3.1-Flag-DVL2 and pcDNA3.1-Flag-DVL3) for 72 h, were lysed to examine expressions of GSK3β, P-GSK3β (Ser9), β-catenin and DVL1-3 using Western blotting. (**B**) and (**C**) The GSK3β inhibitor Licl did not control the accumulation of β-catenin in cytoplasm and nucleus. HCT-8 cells treated with Licl (20 mM) for 72 h， were lysed to examine cytoplasmic and nuclear protein levels of GSK3β, P-GSK3β (Ser9) and β-catenin using Western blotting. In each case, the blot is representative of immunoblots resulting from three separate experiments.
